# The mechanism of filler items in the response time concealed information test

**DOI:** 10.1007/s00426-020-01432-y

**Published:** 2021-01-15

**Authors:** Gáspár Lukács, Ulrich Ansorge

**Affiliations:** 1 Department of Cognition, Emotion, and Methods in Psychology, Faculty of Psychology, University of Vienna, Liebiggasse 5, 1010 Vienna, Austria; 2 Vienna Cognitive Science Hub, University of Vienna, Liebiggasse 5, 1010 Vienna, Austria

## Abstract

The response time concealed information test (RT-CIT) can reveal that a person recognizes a relevant (probe) item among other, irrelevant items, based on slower responding to the probe compared to the irrelevant items. Therefore, if this person is concealing the knowledge about the relevance of this item (e.g., recognizing it as a murder weapon), this deception can be unveiled. Adding familiarity-related filler items to the task has been shown to substantially increase the validity of the method, but assumptions for this effect have never been tested before. In the present series of three experiments (*N* = 511), we tested several factors, most of which were found to indeed influence the enhancing effects of fillers. First, larger enhancement is achieved when a smaller proportion of fillers shares the response key with the target. Second, familiarity context does play a role in the enhancement, and the target sharing its response key with the familiarity-referring fillers leads to larger enhancement. Third, mere symbolic fillers (such as simple arrow-like characters) also lead to enhancement, but filler words without task-relevant meaning are not effective. Fourth, small visual differences (lettercase or underlining) between fillers and the rest of the items have no significant influence. All this provides justification for the original structure of the fillers and also demonstrates that the enhancement is highly generalizable: Fillers have a potential to improve the RT-CIT regardless of deception scenario, item types, or the examinee's language comprehension.

## Introduction

Undetected deception may lead to extreme costs in certain scenarios such as counterterrorism, pre-employment screening for intelligence agencies, or high-stakes criminal proceedings. However, meta-analyses have repeatedly shown that without special aid, based on their own best judgment only, people (including police officers, detectives, and professional judges) distinguish lies from truths on a level hardly better than mere chance (Bond & DePaulo, 2006; Hartwig & Bond, 2011; Kraut, 1980). Therefore, researchers have advocated special techniques that facilitate lie detection, including computerized tasks such as the concealed information test (CIT; Lykken, 1959; Meijer, Selle, Elber, & Ben-Shakhar, 2014).

The CIT aims to disclose whether examinees recognize certain relevant items, such as a weapon used in a recent homicide, among a set of other objects, when they actually try to conceal any knowledge about the criminal case. In the response time (RT)-based CIT, participants classify the presented stimuli as the target or as one of several nontargets by pressing one of two keys (Seymour, Seifert, Shafto, & Mosmann, 2000; Suchotzki, Verschuere, Van Bockstaele, Ben-Shakhar, & Crombez 2017; Varga, Visu-Petra, Miclea, & Buş, 2014). Typically, five nontargets are presented, among which one is the *probe*, which is an item that only a guilty person would recognize, and the rest are *irrelevants*, which are in most respects (e.g., their category membership) similar to the probe and, thus, indistinguishable for an innocent person. For example, in a murder case where the true murder weapon was a knife, the probe could be the word "knife," while irrelevants could be "gun," "rope," etc. Assuming that the innocent examinees are not informed about how the murder was committed, they would not know which of the items is the probe. The items are repeatedly shown in a random sequence, and all of them have to be responded to with the same response keys, except one arbitrary *target*—a randomly selected, originally also irrelevant item that has to be responded to with the other response key. Since guilty examinees recognize the probe as a relevant item, too, it will become unique among the irrelevants and in this respect more similar to the rarely occurring target (Lukács & Ansorge, 2019a). Due to this conflict between instructed response classification of probes as nontargets on the one hand, and the probe's uniqueness and, thus, greater similarity to the alternative response classification as potential target on the other hand, the response to the probe will be generally slower in comparison to the irrelevants (Seymour & Schumacher, 2009). Consequently, based on the probe-to-irrelevant RT differences, guilty (i.e., knowledgeable) examinees can be distinguished from innocent (i.e., naive) examinees.

A recent study significantly improved the RT-CIT (i.e., significantly increased the accuracy of distinguishing guilty examinees from innocent ones) by adding familiarity-related filler items to the task (Lukács, Kleinberg, & Verschuere, 2017b). The paper described several hypotheses to explain why the fillers improved the RT-CIT. However, none of these hypotheses were tested in the study, which merely demonstrated that the addition of filler items indeed increased the classification accuracy of the RT-CIT. The present study aims to test the key hypotheses, as well as some potentially relevant underlying factors, and, thereby, gain insight into the mechanism of filler items in the RT-CIT.

### Semantic context

The inclusion of filler items was originally inspired by the Implicit Association Test (IAT; Bluemke & Friese, 2008; Greenwald, McGhee, & Schwartz, 1998; Karpinski & Steinman, 2006; Nosek, Greenwald, & Banaji, 2007; see also: Agosta & Sartori, 2013; Lukács, Gula, Szegedi-Hallgató, & Csifcsák, 2017a; Verschuere & De Houwer, 2011). The IAT measures the strength of associations between certain critical items to be discriminated, such as concepts or entities (e.g., various political parties), and certain attribute items to be evaluated (e.g., positive vs. negative words). The main idea is that responding is easier (and thus faster) when items closely related in their subjective evaluation share the same response key (Greenwald et al., 2009; Nosek et al., 2007). For example (taken from Bluemke & Friese, 2008), a person with an implicit preference for a specific political party responds faster when having to categorize stimuli related to that party (e.g., party emblems or names of well-known party members) together with positive words (e.g., joy, health). Inversely, the categorization of the same stimuli (for the preferred party) will be slower when they share a response key with negative words (e.g., pain, disease).

It was assumed that an analogous mechanism may be introduced in the CIT by adding probe-referring “attributes,” that is, filler items in the task. In the original study (Lukács et al., 2017b), the probes were certain personal details of the participants (their birthday, favorite animal, etc.), which were, therefore, “familiar” (self-related, recognizable, etc.) to the given participant, as opposed to the irrelevants (e.g., other dates, random animal names) that were in this respect relatively “unfamiliar” (other-related, etc.). Two corresponding kinds of fillers were added to the task: (a) familiarity-referring words (“FAMILIAR,” “RECOGNIZED,” and “MINE”) that had to be categorized with the same key as the target (and, thus, with the opposite key than the probe and the irrelevants), and (b) unfamiliarity-referring words (“UNFAMILIAR,” “UNKNOWN,” “OTHER,” “THEIRS,” “THEM,” and “FOREIGN”) that had to be categorized with the same key as the probe (and irrelevants). It was assumed that this would have a similar effect as in the IAT: Reponses to the self-related probes (true identity details) would be even slower because they have to be categorized *together* with other-referring expressions (and opposite to self-referring expressions). In contrast, in case of innocents, the probes are not self-related; hence, the fillers will not slow down the responses to the probe further.

### Task complexity

The other key assumption described in the paper (Lukács et al., 2017b) was that the increased task difficulty due to the increased task complexity required more attention throughout the task, which likely facilitated deeper processing of the stimuli (Lukács et al., 2017b, p. 3). Task difficulty may also be conceptualized as or reflected in cognitive load (e.g., Suchotzki et al., 2017). At least two previous experiments reported that increased cognitive load increases probe-irrelevant differences (Hu, Evans, Wu, Lee, & Fu, 2013; Visu-Petra, Varga, Miclea, & Visu-Petra, 2013, see also Visu-Petra, Miclea, & Visu-Petra 2012)—although it may be added that some factors potentially related to cognitive load (e.g., speed instructions, pace of presentation) assessed in a recent meta-analysis were not found to be contributing factors in RT-based deception research in general (though not necessarily in CIT specifically; Suchotzki et al., 2017). In any case, there is repeated evidence that more complex RT-CIT designs lead to larger probe-irrelevant differences (Hu et al., 2013; Verschuere, Kleinberg, & Theocharidou, 2015; Visu-Petra et al., 2013).

We have at least one specific idea why increased complexity could be beneficial. In a CIT without filler items (and with a single probe and single target; Verschuere et al., 2015), there is a single item, the target, that requires a different response key as opposed to all the other items. This allows participants to focus attention to that single target item: For example, participants can perform the task by the rule of pressing Key *I* in case of a target, and pressing Key *E* in case of anything else (cf. Verschuere et al., 2015). In our view, this is not necessarily a conscious decision: This is how the task and its associated stimulus probabilities are represented mentally, because humans are sensitive to different stimulus probabilities and novel information for learning and efficient processing (cf., e.g., Anderson, 1991; Kim, 2014; Parmentier, Elford, Escera, Andrés, & San Miguel 2008; Reber, 1989). This is reflected in slower responses to targets, higher error rates to targets, and higher P300 brainwave responses to targets as compared to the irrelevants (Farwell & Donchin, 1991; Rosenfeld, Biroschak, & Furedy 2006, 2004; Wasserman & Bockenholt, 1989). In sum, we assume that participants pay most of their attention to this single item, and to some extent ignore the rest. They categorize each item as target or “not the target.” Thereby, participants also ignore (to some extent) the meaning of the probe, and, accordingly, the difference in meaning between probe and irrelevants. That is, when the probe appears, participants just register that it is a “nontarget” (perhaps already from visual differences, e.g., seeing the starting letter which is different from the target’s starting letter), and they hardly even recognize its meaning (and thereby its task relevance, which would be the essence of a CIT effect).

Now, as soon as we add more items, the task ceases to be that simple. When there are more items to categorize, participants cannot anymore go by the rule of “the single target item or anything else.” There are multiple items to be categorized with both response keys (i.e., including the target’s *I*-Key response). Hence, even if participants again focus on target-category items (e.g., “pressing Key *I* for the target *or* one of the three fillers, pressing Key *E* for anything else”), this will not be so easy to do. Participants have to pay much more attention to which item requires which response. Sometimes a target comes that needs Key *I* to be pressed, but sometimes some other item requires this response, so participants cannot just focus on the target. Consequently, they have to process more deeply each item that appears. Hence, when the probe appears, participants process that more deeply, too, and they cannot easily ignore its meaning. They are then more inclined to recognize it as an item meaningfully related to the task, which in turn elicits response conflict, leading to slower responses and a larger CIT effect.

### Proportion

Finally, one assumption was not explicitly mentioned in the original paper because it does not directly influence the validity of the RT-CIT, but concerns the arrangement of the fillers themselves. Namely, the smaller proportion (3–6) of familiarity-referring relative to unfamiliarity-referring items used in the study was intended to keep “target-category” items (i.e., the target and items to be categorized with the same key as the target) less frequent than the rest of the items, thereby, keeping the target (or all target-side items) more rare or even unique among all used items. This uniqueness (or “pop-out”) of target-category items is assumed to be a factor in eliciting response conflict in case of, in this respect similar, probes among the irrelevant items (Lukács & Ansorge, 2019a; Seymour & Schumacher, 2009), thereby, contributing to larger probe-irrelevant differences.

We described the effects of mapping semantic concepts to response keys as exemplified by the IAT. However, adverse effects of feature overlap on categorization are more general. Categorization is generally most efficient in cases where “most attributes [are] common to members of the category and the least attributes shared with members of other categories” (Rosch, Mervis, Gray, Johnson, & Boyes-Braem 1976, p. 1435; see also, e.g., Iordan, Greene, Beck, & Fei-Fei, 2016). Overlapping features can be as simple as visual cues (e.g., Azizian, Freitas, Watson, & Squires 2006; Marchand, Inglis-Assaff, & Lefebvre 2013). Yet, pertinent to uniqueness (and, hence, proportion) in the context of the present study, it has been demonstrated that stimulus categorization can be based on differences in item salience as well (Rothermund & Wentura, 2004): When both high-salient and low-salient items have to be categorized, responding is easier (and, thus, faster) when items of similar salience share the same response key (i.e., one response key for high-salient items, another for low-salient ones).

In this context, uniqueness or rarity of a stimulus can be regarded as a form of salience itself. Just as, for example, visual distinctness in terms of features of items across space creates a form of salience (e.g., a single red item is salient if presented together with green items; Itti & Koch, 2000), rare or surprising information across time is salient (e.g., a single red item is salient if presented in a sequence of green items) and, for example, captures attention: such rarity or surprisingness is a mediating factor of the influence of visual distinctness across space itself (Horstmann, 2002, 2005; Itti & Baldi, 2009). In the standard CIT (without fillers), the target shares the semantic category of the irrelevant and probe items (e.g., dates in case of looking for a birthday), and its only distinction is that it is the single item that requires a different key response,^1^ which makes it unique or salient (in terms of its associated response meaning) among the rest of the items, and, also, a relevant item in the task. The only other item of a unique meaning and, thus, also relevant in the task by this uniqueness among the stimuli, is the probe. Hence, the probe, as opposed to the irrelevants, will share the target's feature of uniqueness or salience, and, accordingly, of relevance, and the probe will, therefore, be more difficult to categorize together with the irrelevants.

Looking at this via the theoretical framework of polarity correspondence (Proctor & Cho, 2006), examinees probably code targets as + pole items and irrelevants as − pole items along the dimension of task relevance. If participants assign similar + poles versus − poles to less frequent versus more frequent items, then classification of rare targets would benefit from polarity correspondence, meaning that these items would be positive on two poles: relevance and uniqueness. Accordingly, classification of probes—which are rare among the irrelevants—would suffer from a double polarity non-correspondence, being of a different polarity in terms of two dimensions from the rest of the irrelevant items, with which the probes are to be categorized.

Finally, as closely related empirical evidence, one previous RT-CIT study already demonstrated that 1:1:1 proportions of target:probe:irrelevant, as opposed to the conventional 1:1:4 proportions, robustly decreased (and reversed) probe-irrelevant RT differences (Suchotzki, Verschuere, Peth, Crombez, & Gamer, 2015). This result alone allows other interpretations as well (e.g., the authors of that paper pointed more toward the proportion of probe vs. irrelevants, rather than the proportion of target vs. nontargets), but still the finding is clearly in line with our assumption.

Altogether, we have good reason to think that the rarity of the target—and, by extension, the rarity of all target-category items—is an important (if not a key) factor in eliciting probe-irrelevant differences. Eliminating the rarity of target-side items (and, thereby, the association of rarity with the response key opposite to the key for probe) simultaneously eliminates the importance and influence of the probe’s rarity among the irrelevants, and it is thereby expected to lead to diminished probe-irrelevant differences.

### Distinctness

Somewhat relatedly, in the present study, we also examined whether visually more distinct fillers (e.g., lowercase fillers as opposed to uppercase probe, target, and irrelevant items) undermine the assumed boosting effects of semantic relatedness, of elaboration, or even of proportions on the probe-irrelevant differences (Lukács & Ansorge, 2019b; Lukács, Grządziel, Kempkes, & Ansorge 2019). First, if a visual distinction separates the two tasks ([a] categorization of fillers vs. [b] categorization of the rest of the items) from the participants’ perspective, it could also undermine the conceptual relation of the two tasks (cf. Craik & Lockhart, 1972) and the proportion of the fillers (which is, thus, less related to the target-nontarget discrimination task with probe, irrelevants, and target items) should matter less for the probe-irrelevant difference: That is, a distinctness × proportion interaction would be found, with smaller differences between different proportions when the fillers are visually distinct (presented in lowercase). Second, instead, the increased visual distinction may also draw more attention to the semantic distinction and lead to more salience of each of the two separate categories (again: [a] fillers and [b] other items), and, thereby, increase probe versus irrelevant performance differences’ effect sizes.

### Study structure

In the first experiment, we tested the effect of proportions, along with the effect of distinctness (which we thought might interact with the Proportion effect). In the second experiment, we tested the effect of semantic context, along with further testing of distinctness. The third experiment tested the effect of task complexity. This third experiment gave some unexpected results, and was, therefore, conceptually replicated with different stimuli for confirmation in Experiment 3b.

All statistical tests followed our corresponding preregistrations (Exp. 1: https://osf.io/6e3bz; Exp. 2: https://osf.io/ju845; Exp. 3a: https://osf.io/b4ghe; Exp. 3b: https://osf.io/rsguj; Foster & Deardorff, 2017; Wagenmakers, Wetzels, Borsboom, van der Maas, & Kievit, 2012). Supplementary, not preregistered tests were added in the Appendix 1.

## Experiment 1

To test the effect of proportion, we compared the *Original*, “3–6” (familiar-referring item number to unfamiliar-referring item number) version with a version with a *Reverse* proportion “6–3” (familiar-referring item number to unfamiliar-referring item number), which accordingly included six familiar-referring items unlike all previous versions (these were: “FAMILIAR,” “RECOGNIZED,” “MINE,” “RELEVANT,” “MEANINGFUL,” “KNOWN”; while the six unfamiliarity-referring words were: “UNFAMILIAR,” “IRRELEVANT,” “OTHER,” “THEIRS,” “RANDOM,” “FOREIGN”). In each condition, the three items for the smaller group of stimuli were chosen randomly, for each participant, out of the full set of six. To test the effect of distinctness, we simply displayed fillers in lowercase (*Distinct* condition) as opposed to uppercase (*Regular* condition), while the rest of the items (probes, targets, irrelevants) always remained uppercase as conventional.^2^ We used a within-subject design, with each participant tested with both proportion conditions (original or reverse) as well as both distinctness conditions (distinct or regular); see procedure. All participants were tested with their own country of origin as probe in the CIT task, simulating a guilty suspect trying to conceal the recognition of this country name.

### Methods

#### Participants

This experiment was run on Figure Eight (https://www.figure-eight.com; formerly known as CrowdFlower), an online crowdsourcing platform where participants from anywhere in the world can register to complete small online tasks (Peer, Samat, Brandimarte, & Acquisti, 2015). Hence, this website may also be used to offer participation in online experiments by providing a link to the task to be completed (e.g., Kleinberg & Verschuere, 2015). People registered on this site as “contributors” complete many such tasks, and their performance may be rated after the completion of the tasks by the “customers” who offered those tasks. Based on these ratings, contributors are categorized into three levels, where contributors with best ratings are categorized as “Level 3.” When creating a new task, a customer (in this case, the current authors) may choose the lowest level of contributors that are allowed to take the task. We set this to “Level 3”, hence, only such “Level 3” contributors were allowed to participate in the study. We paid 1.20 USD per completed task, which took about 20 min. The task could only be completed in one uninterrupted time from one IP address: Another attempt from an IP address that was already stored with a completed task resulted in a warning prompt on the first page of the task that did not allow continuation.

We initially opened 60 slots, and afterwards opened 30 additional slots three times due to not having reached *BF* = 5 for the main analysis of variance’s (ANOVA’s) interaction (see preregistration). Eventually, altogether 157 participants completed the test, but due to an unfortunate temporary server issue 17 were not saved. Hence, we obtained 140 complete CIT data samples.

Our exclusion criteria were at least 50% accuracy for each of the following item categories: targets, self-referring fillers, other-referring fillers. Furthermore, at least 75% overall accuracy for main items (probe or irrelevant items). Only one participant had to be excluded based on these criteria. However, a further six participants were excluded due to not recalling correctly the probes at the end of the task (see “Procedure”). This left 133 participants (*M*_age_ ± SD_age_ = 35.5 ± 10.1, 94 male).

#### Procedure

Before beginning the experiment, all participants agreed to the informed consent to proceed further. Participants then provided demographic information, including their country of origin. Participants were then informed that the following task simulates a lie detection scenario, during which they should try to hide their country of origin. They were then presented a short list of randomly chosen country names. The country names on this list did not contain the probe (the true country of origin of a given participant), but they had the closest possible character length to the given probe, none of them started with the same letter, and if the probe included a space (e.g., “New Zealand” or “Czech Republic”), the items on this list were all chosen to include a space as well. The participants were asked to choose any (but a maximum of two) country names that were personally meaningful to them or in any way appeared different from the rest of the items on these lists. Subsequently, five country names for the CIT were randomly selected from the non-chosen items (as this assures that the irrelevants were indeed irrelevant). One of these items was randomly chosen as the target, while the remaining four served as irrelevants.

During the RT-CIT, the items were presented one by one in the center of the screen, and participants had to categorize them by pressing one of two keys (*E* or *I*) on their keyboard. They had to press Key *I* whenever a target or a familiarity-referring filler appeared, while they had to press Key *E* whenever the probe, an irrelevant, or an unfamiliarity-referring filler appeared. The inter-trial interval (i.e., between the end of one trial and the beginning of the next) always randomly varied between 300 and 600 ms. In case of a correct response, the next trial followed. In case of an incorrect response or no response within the given time limit, the caption “WRONG” or “TOO SLOW” in red color appeared, respectively, below the stimulus for 300 ms, followed by the next trial.

The main task was preceded by three practice tasks. In the first practice task, all filler items were presented twice, hence, altogether 18 items. In case of few valid responses, the participants received a corresponding feedback, were reminded of the instructions, and had to repeat the practice task. The requirement was a minimum of 80% valid responses (i.e., pressing of the correct key between 150 and 900 ms following the display of an item) for each of the two filler types.

Next, participants were presented with their targets and were asked to memorize them to recognize them as requiring a different response during the following task. On the next page, participants were asked to recall the memorized targets and could proceed only if they selected these targets correctly from a dropdown menu. If the entered item was incorrect, the participant received a warning and was redirected to the previous page to have another look at the same items.

Then the second practice task followed, in which all items (nine fillers, one probe, one target, four irrelevants) were presented once, and participants had plenty of time (10 s) to choose a response. However, each trial required a correct response. In case of an incorrect response, the participant immediately got a corresponding feedback, was reminded of the instructions, and had to repeat this practice. This guaranteed that the eventual differences (if any) between the responses to the probe and the responses to the irrelevants were not due to misunderstanding of the instructions or any uncertainty about the required responses in the eventual task.

In the third and final practice task, again all items were presented once, but the response deadline was again short (900 ms) and a certain rate of mistakes were again allowed. In case of few valid responses, the participants received a corresponding feedback, were reminded of the instructions, and had to repeat the practice task. The requirement was a minimum of 60% valid responses (pressing of the correct key between 150 and 900 ms following an item) for each of the following item types: familiarity-referring filler, unfamiliarity-referring filler, target, or main items (probe or irrelevants together).

The main task, in each test, contained four blocks. The first two blocks both had either original proportion or reverse proportion of fillers; whereas the last two blocks both had the opposite type of proportion for the given participant. The distinctness condition alternated per each block: for instance, first distinct, then regular, then again distinct, then again regular—otherwise the reverse (first regular, then distinct, etc.). The orders (for either factor) were assigned randomly.

In each block, each probe, irrelevant, and target was repeated 18 times (hence, 18 probe, 72 irrelevant, and 18 target trials, in each block). The order of these items was randomized in groups: first, all six items (one probe, four irrelevants, and one target) in the given category were presented in a random order, then the same six items were presented in another random order (but with the restriction that the first item in the next group was never the same as the last item in the previous group). Fillers were placed among these items in a random order, but with the restrictions that a filler trial was never followed by another filler trial, and each of the nine fillers preceded each of the other items (probes, targets, and irrelevants) exactly one time. (Thus, 9 × 6 = 54 fillers were presented per block, and 54 out of the 108 other items were preceded by a filler).

At the end of the test, to verify the proper understanding of the lie detection scenario simulation and the genuineness of the country of origin, each participant had to select their true country of origin (i.e., the probe) from the list of all possible countries. Participants who selected the country incorrectly were excluded from the analyses (Lukács & Ansorge, 2019a). Finally, the participants were given a brief explanation about the purpose of the study and contact details.

#### Data analysis

For examining the main questions, the dependent variable was always the probe-irrelevant correct RT mean (probe RT mean minus irrelevant RT mean, per each participant, using all valid trials). As secondary analyses, we also report all tests with (a) accuracy rates (ratio of correct responses to the sum of correct, incorrect, and too slow responses) and (b) keypress durations, in place of RT means. In a recent study, keypress durations were found to be shorter for probe responses as compared to irrelevants, thereby providing an additional possible index of the CIT effect (Lukács, Kleinberg, Kunzi, & Ansorge, 2020). Therefore, it seemed interesting to report it here too. Nonetheless, RT is always the primary measure in the RT-CIT.

We report Bayes factors (*BF*s) using the default *r*-scale of 0.707 (Morey & Rouder, 2018). In case of ANOVAs, we report inclusion *BF*s based on matched models (Makowski, Ben-Shachar, & Lüdecke 2019; Mathôt, 2017). The *BF* is a ratio between the likelihood of the data fitting under the null hypothesis and the likelihood of fitting under the alternative hypothesis (Jarosz & Wiley, 2014; Wagenmakers, 2007). For example, a Bayes factor (*BF*) of 3 means that the obtained data are three times as likely to be observed if the alternative hypothesis is true, while a *BF* of 0.5 means that the obtained data are twice as likely to be observed if the null hypothesis is true. Here, for more readily interpretable numbers, we denote Bayesian factors as *BF*_*10*_ for supporting the alternative hypothesis, and as *BF*_*01*_ for supporting null hypothesis. Thus, for example, *BF*_*01*_ = 2 again means that the obtained data are twice as likely under the null hypothesis than under the alternative hypothesis. Typically, *BF* = 3 is interpreted as the minimum likelihood ratio for “substantial” evidence for either the null or the alternative hypothesis (Jeffreys, 1961).

To calculate illustrative areas under curves (AUCs) for probe-irrelevant RT mean differences as predictors, we simulated control groups for the RT data from each of the four possible conditions, using 1,000 normally distributed values with a mean of zero and an *SD* derived from the real data (from each condition) as *SD*_real_ × 0.5 + 7 ms (which has been shown to very closely approximate actual AUCs; Lukács & Specker, 2020; the related function is available in the analysis codes uploaded to the OSF repository). We would like to emphasize that these simulated AUCs are just approximations for illustration, and we do not use them for any of our statistical tests.

To demonstrate the magnitude of the observed effects, for *F* tests, we report generalized eta squared (*η*_G_^2^) and partial eta squared (*η*_p_^2^) with 90% CIs (Lakens, 2013). We report Welch-corrected *t* tests (Delacre, Lakens, & Leys, 2017), with corresponding Cohen’s *d* values as standardized mean differences and their 95% CIs (Lakens, 2013). We used the conventional alpha level of 0.05 for all statistical significance tests.

For all analyses, RTs below 150 ms were excluded. For RT analyses, only correct responses were used. For keypress duration analysis only, keypress durations not below 200 ms were excluded.^3^ Accuracy was calculated as the number of correct responses divided by the number of all trials (after the exclusion of those with an RT below 150 ms). All analyses were conducted in R (R Core Team, 2019; via: Kelley, 2019; Lawrence, 2016; Makowski et al., 2019; Morey & Rouder, 2018).

### Results

Aggregated means of RT means, accuracy rates, and keypress durations, for the different stimulus types in each condition, are given in Table 1.

**Table 1:** Reaction time (RT) means, accuracy rates, and keypress durations, in Experiment 1

	RT mean		Accuracy rate		Keypress duration	
	Original	Reverse	Original	Reverse	Original	Reverse
*Regular*						
Probe	532 ± 64	552 ± 61	97.4 ± 5.7	96.2 ± 6.3	128 ± 28	128 ± 25
Irrelevant	505 ± 48	534 ± 56	99.2 ± 2.4	98.2 ± 3.7	128 ± 28	128 ± 26
Target	602 ± 50	606 ± 56	86.3 ± 11.6	85.3 ± 11.5	124 ± 28	123 ± 27
Filler-F	620 ± 48	607 ± 49	84.9 ± 11.5	89.7 ± 7.9	123 ± 28	123 ± 27
Filler-U	569 ± 58	613 ± 76	94.8 ± 6.5	86.4 ± 13.7	127 ± 27	127 ± 25
P–I	27.9 ± 43.4	17.9 ± 36.2	− 1.81 ± 4.78	− 2.00 ± 6.60	0.0 ± 5.9	0.1 ± 4.8
*Distinct*						
Probe	522 ± 61	543 ± 60	98.7 ± 3.1	96.9 ± 6.9	129 ± 28	127 ± 26
Irrelevant	498 ± 47	524 ± 56	99.5 ± 1.0	98.4 ± 2.2	129 ± 28	127 ± 27
Target	598 ± 45	609 ± 55	84.0 ± 12.2	84.0 ± 12.4	125 ± 28	123 ± 27
Filler-F	614 ± 48	595 ± 47	86.6 ± 11.7	91.8 ± 9.1	124 ± 27	122 ± 28
Filler-U	571 ± 58	623 ± 74	94.7 ± 5.6	83.8 ± 14.0	128 ± 27	126 ± 26
P–I	23.5 ± 40.4	19.0 ± 35.0	− 0.84 ± 3.10	− 1.46 ± 6.89	0.5 ± 4.5	0.1 ± 5.2

Means and SDs (in the format of *M* ± SD) for individual RT means, accuracy rates, and keypress durations; for *probe* (participants’ own countries), *irrelevant* (other countries), *target* (the designated irrelevant details that require different response), *P–I* (individual probe minus irrelevant values); per each experimental condition (factors proportion [original vs. reverse] and distinctness [regular vs. distinct])

*F* familiarity referring (target side), *U* unfamiliarity referring (nontarget side)

#### RT means

The proportion main effect was significant, with strong evidence for larger probe-irrelevant RT mean differences in the original condition than in the reverse condition, as expected: *F*(1, 132) = 9.85, *p* = 0.002, *η*_p_^2^ = 0.069, 90% CI [0.016, 0.147], *η*_G_^2^ = 0.009, *BF*_*10*_ = 49.14. There was, however, no distinctness main effect and the distinctness × proportion interaction, with *BF*s supporting equivalence; *F*(1, 132) = 0.73, *p* = 0.396, *η*_p_^2^ = 0.005, 90% CI [0, 0.044] (main effect), *η*_G_^2^ < 0.001, *BF*_*01*_ = 7.36; *F*(1, 132) = 2.40, *p* = 0.123, *η*_p_^2^ = 0.018, 90% CI [0, 0.071], *η*_G_^2^ = 0.001, *BF*_*01*_ = 3.29 (interaction). This means that more distinct fillers (i.e., ones displayed in lowercase as opposed to the rest of the items in the task) had no significant influence on the outcomes.

#### Accuracy rates

There were no significant main effects nor interaction for probe-irrelevant accuracy rate differences. Proportion main effect: *F*(1, 132) = 1.31, *p* = 0.255, *η*_p_^2^ = 0.010, 90% CI [0, 0.055], *η*_G_^2^ = 0.001, *BF*_*01*_ = 6.50; distinctness main effect: *F*(1, 132) = 3.81, *p* = 0.053, *η*_p_^2^ = 0.028, 90% CI [0, 0.088], *η*_G_^2^ = 0.005, *BF*_*01*_ = 2.16; distinctness × proportion interaction: *F*(1, 132) = 0.18, *p* = 0.671, *η*_p_^2^ = 0.001, 90% CI [0, 0.029], *η*_G_^2^ < 0.001, *BF*_*01*_ = 6.90.

#### Keypress durations

There were also no significant main effects or interaction for probe-irrelevant keypress duration differences. Proportion main effect: *F*(1, 129) = 0.08, *p* = 0.781, *η*_p_^2^ = 0.001, 90% CI [0, 0.023], *η*_G_^2^ < 0.001, *BF*_*01*_ = 9.92; distinctness main effect: *F*(1, 129) = 0.22, *p* = 0.643, *η*_p_^2^ = 0.002, 90% CI [0, 0.031], *η*_G_^2^ < 0.001, *BF*_*01*_ = 9.35; distinctness × proportion interaction: *F*(1, 129) = 0.49, *p* = 0.486, *η*_p_^2^ = 0.004, 90% CI [0, 0.040], *η*_G_^2^ = 0.001, *BF*_*01*_ = 6.35.^4^

#### AUCs

The simulated AUCs for probe-irrelevant RT mean differences were as follows. Original proportion: 0.684, 95% CI [0.633, 0.736] (regular) and 0.666, 95% CI [0.614, 0.718] (distinct); reverse proportion: 0.641, 95% CI [0.583, 0.699] (regular) and 0.655, 95% CI [0.604, 0.706] (distinct).

## Experiment 2

The second experiment aims primarily at testing the effect of semantic context, although it also involves further testing of distinctness. The semantic relevance of fillers was tested by switching the required response keys for familiarity-referring versus unfamiliarity-referring fillers. That is, one condition was as usual (familiarity-referring fillers categorized together with targets, unfamiliarity-referring fillers opposite of targets; probe-incompatible mapping of fillers), but in another condition, the familiarity-referring fillers had to be categorized together with the probe and irrelevant items, while unfamiliarity-referring fillers had to be categorized together with the target (reversed, probe compatible mapping of fillers). This shifted probe categorization to be in line with the filler categorization: guilty examinees should find it easier to categorize the probe with the key that is also used for categorizing familiarity-referring fillers. In other words, the filler mapping became probe-compatible. Faster responses to probes lead to decreased probe-irrelevant RT differences, hence, decreasing the size of the probe-irrelevant difference and the CIT effect.

This would prove two basic assumptions at once: that (a) meaning of the familiarity-related fillers matters, and (b) key mapping (i.e., “direction” of filler categorization) matters, too. Without proving the second part (e.g., instead of switching key mapping, using neutral, task-unrelated fillers in place of familiarity-related fillers), one may still argue that the mere presence of familiarity-related items has an effect. Since switching of response keys mid-task might be confusing for a participant and could, therefore, affect the second half of the test, to avoid such carry-over effects this factor was tested between-subjects, in two groups (probe incompatible and probe compatible).

Additionally, we conducted further tests on distinctness: In Experiment 1, the fillers changed between uppercase and lowercase, while here the main items (probe, irrelevants, target) changed between uppercase and lowercase (while fillers always remained uppercase). In addition, to further increase visual distinctness, fillers were either underlined or not underlined, in different blocks. This factor was tested within subject, in the conventional Probe-Incompatible group (since we had anticipated that the probe-compatible group would yield robustly decreased probe-irrelevant differences, hence, would not be sensitive for testing any other modulating effects).

We were not interested in the interaction between these two factors: The experiment includes both factors at the same time simply to be economical. Since one factor was tested within subject, and the other between subjects, they could not interfere with each other.

### Methods

The methods of Experiment 2 were identical to those of Experiment 1, except for the details described below.

#### Participants

We initially opened 120 slots on Figure Eight, and 127 participants completed the task. At that point, the *BF* for the between-subjects test of semantic context effect already passed 5, but not the within-subject ANOVA testing distinctness effects in the probe-incompatible condition (see preregistration and Results below). Therefore, we opened 30 additional slots three times in this condition only. Altogether 218 participants completed the test.

Three participants had to be excluded due to too low accuracy rates. An additional 11 participants were excluded due to not recalling correctly the probes at the end of the task. This left 204 participants, 145 in the probe-incompatible group (*M*_age_ ± SD_age_ = 34.5 ± 9.0, 94 male) and 59 in the probe-compatible group (*M*_age_ ± SD_age_ = 33.7 ± 9.7, 33 male).

#### Procedure

In all tests in this experiment, the proportion of fillers was the same: three fillers categorized together with target, six fillers categorized opposite of target. There was a fixed group of six words for familiarity-referring and for unfamiliarity-referring fillers, same as in Experiment 1. In case of fillers categorized opposite of target, all six fillers were used from the given type: unfamiliarity-referring fillers in the probe-incompatible group, and familiarity-referring fillers in the probe-compatible group. In case of fillers categorized together with target, three fillers were randomly from the given type: familiarity-referring fillers in the probe-incompatible group, and unfamiliarity-referring fillers in the probe-compatible group.

The main task, in each test and both groups, again contained four blocks. The first two blocks both had fillers either all underlined or all not underlined; whereas the last two blocks both had either fillers all not underlined or all underlined, respectively, for the given participant. The lettercase of fillers alternated per each block: for instance, first lowercase, then uppercase, then again lowercase, then again uppercase—otherwise the reverse (first uppercase, then lowercase, etc.). The orders (for either factor) were assigned randomly.

### Results

Aggregated means of individual RT means, accuracy rates, and keypress durations, for the different stimulus types in the probe-incompatible and probe-compatible conditions, are given in Table 2. (An extensive supplementary table for the results, in the probe-incompatible group, per visual distinctness conditions—with no significant differences—was uploaded to https://osf.io/f8z4t/.)

**Table 2:** Reaction time (RT) means, accuracy rates, and keypress durations, in Experiment 2

	RT mean		Accuracy rate		Keypress duration	
	P.-Incomp	P.-Comp	P.-Incomp	P.-Comp	P.-Incomp	P.-Comp
Probe	519 ± 70	499 ± 54	98.3 ± 2.5	98.8 ± 2.8	126 ± 29	131 ± 31
Irrelevant	497 ± 55	497 ± 49	98.9 ± 1.7	98.9 ± 1.5	125 ± 30	131 ± 32
Target	598 ± 53	599 ± 53	82.9 ± 9.6	80.8 ± 12.2	122 ± 29	132 ± 30
Filler-F	614 ± 54	620 ± 53	83.6 ± 9.9	80.8 ± 10.8	122 ± 28	131 ± 29
Filler-U	558 ± 65	556 ± 55	94.8 ± 4.6	96.3 ± 2.8	125 ± 29	130 ± 31
P–I	21.8 ± 34.7	1.7 ± 26.2	− 0.55 ± 2.44	− 0.06 ± 2.11	0.3 ± 3.0	0.5 ± 3.1

Means and SDs (in the format of M ± SD) for individual RT means, accuracy rates, and keypress durations; for *probe* (participants’ own countries), *irrelevant* (other countries), *Target* (the designated irrelevant details that require a different response), *P–I* (individual probe minus irrelevant values); for both Semantic Context conditions (probe incompatible and probe compatible)

*P.* Probe, *Comp.* compatible, *Incomp.* Incompatible, *F* familiarity-referring (target side), *U* unfamiliarity-referring (nontarget-side)

#### RT means

As expected, there were much larger probe-irrelevant RT mean differences in the probe-incompatible group in comparison to those in the probe-compatible group, shown by a Welch-corrected *t* test; *t*(141.2) = 4.50, *p* < 0.001, *d* = 0.62, 95% CI [0.31, 0.93], *BF*_*10*_ = 238.06. There were also no significant main effects or interaction for the ANOVA for the visual distinctness related factors underlining and lettercase in the probe-incompatible group. Underlining main effect: *F*(1, 144) = 0.68, *p* = 0.410, *η*_p_^2^ = 0.005, 90% CI [0, 0.040], *η*_G_^2^ < 0.001, *BF*_*01*_ = 7.07; lettercase main effect: *F*(1, 144) = 2.81, *p* = 0.096, *η*_p_^2^ = 0.019, 90% CI [0, 0.070], *η*_G_^2^ = 0.002, *BF*_*01*_ = 2.81; underlining × lettercase interaction: *F*(1, 144) = 1.38, *p* = 0.242, *η*_p_^2^ = 0.009, 90% CI [0, 0.052], *η*_G_^2^ = 0.001, *BF*_*01*_ = 4.98.

#### Accuracy rates

There were no significant differences found for probe-irrelevant accuracy rate difference between probe-incompatible and probe-compatible conditions, *t*(123.9) = − 1.43, *p* = 0.156, *d* = − 0.21, 95% CI [− 0.51, 0.10], *BF*_*01*_ = 2.61. In probe-incompatible group only; underlining main effect: *F*(1, 144) = 0.12, *p* = 0.726, *η*_p_^2^ = 0.001, 90% CI [0, 0.024], *η*_G_^2^ < 0.001, *BF*_*01*_ = 10.12; lettercase main effect: *F*(1, 144) = 1.99, *p* = 0.161, *η*_p_^2^ = 0.014, 90% CI [0, 0.060], *η*_G_^2^ = 0.002, *BF*_*01*_ = 4.56; underlining × lettercase interaction: *F*(1, 144) = 0.08, *p* = 0.777, *η*_p_^2^ = 0.001, 90% CI [0, 0.021], *η*_G_^2^ < 0.001, *BF*_*01*_ = 8.16.

#### Keypress durations

There were no significant differences found for probe-irrelevant differences between probe-incompatible and probe-compatible conditions: *t*(104.2) =  − 0.50, *p* = 0.616, *d* = − 0.08, 95% CI [− 0.38, 0.23], *BF*_*01*_ = 5.30. In probe-incompatible group only; underlining main effect: *F*(1, 141) = 1.04, *p* = 0.311, *η*_p_^2^ = 0.007, 90% CI [0, 0.047], *η*_G_^2^ = 0.001, *BF*_*01*_ = 6.80; lettercase main effect: *F*(1, 141) = 0.07, *p* = 0.792, *η*_p_^2^ < 0.001, 90% CI [0, 0.020], *η*_G_^2^ < 0.001, *BF*_*01*_ = 10.45; underlining × lettercase interaction: *F*(1, 141) = 0.06, *p* = 0.804, *η*_p_^2^ < 0.001, 90% CI [0, 0.019], *η*_G_^2^ < 0.001, *BF*_*01*_ = 7.57.

#### AUCs

The simulated AUCs for probe-irrelevant RT mean differences were as follows. Probe-incompatible group (all blocks merged): 0.683, 95% CI [0.632, 0.734]; probe-compatible group (all blocks merged): 0.494, 95% CI [0.416, 0.572]. In probe-incompatible group only: Not underlined fillers: 0.689, 95% CI [0.637, 0.742] (uppercase fillers) and 0.646, 95% CI [0.594, 0.697] (lowercase fillers); underlined fillers: 0.655, 95% CI [0.602, 0.708] (uppercase fillers) and 0.645, 95% CI [0.594, 0.697] (lowercase fillers).

## Experiment 3a

To test the effect of increased task complexity alone, we used a CIT version with fillers that were semantically neutral (i.e., not task-relevant in their meaning; not related to deception, recognition, or familiarity) and we compared this version with a version with no fillers at all. We used two kinds of semantically neutral fillers.

First, we used fillers that are meaningful (i.e., denote general concepts) but not related to the recognition (or deception) context of the test. For this, we used animate versus inanimate concepts, which are often used as basic, universal, yet relatively neutral items in categorization tasks (see, e.g., Caramazza & Shelton, 1998). For two easily distinguishable groups, we used a standardized set of items in the specific categories of “four-footed animals” (animate items; e.g., “fox” or “turtle”) and “household furniture” (inanimate items; e.g., “chair” or “lamp”) from a previous paper (VanArsdall, Nairne, Pandeirada, & Cogdill 2015; not CIT related), with the two sets of words matched (by the original authors) for category typicality, concreteness, number of letters, familiarity (sic!), imagery, written frequency, meaningfulness, and relatedness (for details, see VanArsdall et al., 2015).

Second, we used fillers that were not only semantically neutral, but almost completely without meaning: namely, we used varying strings of numbers as items (inspired by a frequently used EEG-based CIT design described in, e.g., Rosenfeld, Hu, Labkovsky, Meixner, & Winograd, 2013), with digits representing smaller numbers categorized with one key, and digits representing larger numbers with the other key. For example, the items “11111,” “2222” had to be categorized with one key, while “8888” and “999999” had to be categorized with the other key (with character lengths [i.e., number of digits] matching those of other given filler items for each participant; see “Procedure” below).

The first type of fillers (animate, inanimate concepts) will be referred to as verbal; while the second type of fillers (number strings) will be referred to as nonverbal. While we assumed that both kinds of fillers increase task complexity and invite elaboration, we further assumed that Nonverbal fillers are very easy to distinguish (twofold: [a] within the filler categories of smaller vs. larger numbers, as well as [b] from the rest of the items in the CIT), hence, pose comparatively less difficulty, while verbal fillers, being somewhat more complex in structure, require more processing, closer attention, and, hence, invite more elaboration and pose comparatively more difficulty.

### Methods

The methods of Experiments 3a and 3b were identical to those of Experiments 1 and 2, except for the details described below.

#### Participants

We opened 60 slots on Figure Eight, and 66 participants completed the task. No participant had to be excluded due to too low accuracy rates. Three participants were excluded due to not recalling correctly the probes at the end of the task. This left 63 participants (*M*_age_ ± SD_age_ = 32.1 ± 9.3, 46 male).

#### Procedure

The main task, in each test, contained three blocks: one block with verbal fillers, one block with nonverbal fillers, and one with no fillers at all (only probe, target, and irrelevant items; still randomized in the same way as in the rest of the blocks, just without additional insertion of fillers).

In the verbal block, the animate and inanimate filler items were, respectively: (a) “bear,” “cat,” “fox,” “mouse,” “rabbit,” “rat,” “sheep,” “tiger,” “turtle,” “wolf”, and (b) “bed,” “cabinet,” “chair,” “couch,” “desk,” “dresser,” “lamp,” “sofa,” “stool,” “table.” The proportion was always “3–6” (three categorized with the same key as the target, six with the other key), with three and six words chosen randomly from the given categories. Categories were assigned randomly to the response keys. That is, for each given participant, it was randomly decided whether animate or inanimate items should be categorized with the same key as the target, while the given other category items always had to be categorized with the opposite key. All items in the CIT were always displayed in uppercase only.

In the nonverbal block, the number string fillers were items consisting of several identical digits, with digits varying from 1 to 9. There were two possible key assignments. In one case, three number strings with digits from 1 to 3 had to be categorized with the same key as target, while six number strings with digits from 4 to 9 had to be categorized with the other key. In the other case, the three number strings with digits from 7 to 9 had to be categorized with same key as the target, while the six number strings with digits from 1 to 6 had to be categorized with the other key. This key assignment was chosen randomly for each participant. In either case, each number string had the same number of digits as the number of characters of a given matched verbal filler in the other block (for a filler categorized with the same key). For example, if a given participant had “fox,” “mouse,” and “wolf” to be categorized together with the target, this participant could have had the number strings “111,” “22222,” and “3333” (or “777,” “88888,” and “9999”) as nonverbal fillers to be categorized together with target.

### Results

Aggregated means of individual RT means, accuracy rates, and keypress durations, for the different stimulus types in each condition, are given in Table 3 (together with the similar data from Experiment 3b).

**Table 3:** Reaction time (RT) means, accuracy rates, and keypress durations, in Experiment 3a and 3b

	RT mean			Accuracy rate			Keypress duration		
	No filler	Nonverbal	Verbal	No filler	Nonverbal	Verbal	No filler	Nonverbal	Verbal
*Exp. 3a*									
Probe	443 ± 50	500 ± 63	498 ± 54	99.2 ± 2.2	98.3 ± 4.6	99.1 ± 2.5	128 ± 27	132 ± 27	132 ± 27
Irrelevant	438 ± 53	479 ± 49	499 ± 45	99.2 ± 1.8	99.2 ± 1.6	99.1 ± 1.2	129 ± 28	132 ± 28	131 ± 28
Target	533 ± 47	595 ± 48	594 ± 50	87.2 ± 10.5	78.8 ± 13.8	81.0 ± 14.1	126 ± 28	129 ± 30	129 ± 34
Filler-T		562 ± 41	585 ± 47		87.0 ± 10.0	82.4 ± 14.3		127 ± 29	126 ± 33
Filler-NT		513 ± 49	517 ± 49		98.1 ± 3.4	97.4 ± 4.0		132 ± 27	132 ± 27
P–I	5.3 ± 33.5	20.6 ± 44.7	− 0.8 ± 35.3	0.01 ± 2.64	− 0.90 ± 4.50	0.06 ± 2.68	− 1.3 ± 5.8	− 0.1 ± 5.0	0.8 ± 4.7
*Exp. 3b*									
Probe	454 ± 72	517 ± 83	520 ± 77	98.2 ± 4.3	97.9 ± 4.8	98.3 ± 4.8	123 ± 27	127 ± 27	127 ± 28
Irrelevant	437 ± 57	489 ± 62	516 ± 60	99.1 ± 2.0	98.5 ± 2.3	98.1 ± 3.7	124 ± 27	127 ± 27	127 ± 28
Target	526 ± 54	589 ± 60	605 ± 59	85.1 ± 11.1	77.5 ± 14.4	80.7 ± 12.0	121 ± 26	122 ± 25	122 ± 26
Filler-T		552 ± 65	622 ± 57		91.7 ± 8.8	79.6 ± 15.2		120 ± 24	123 ± 26
Filler-NT		517 ± 66	547 ± 64		97.5 ± 5.8	94.9 ± 7.0		127 ± 26	127 ± 28
P–I	16.2 ± 33.7	27.5 ± 42.9	3.9 ± 47.0	− 0.93 ± 4.26	− 0.60 ± 4.69	0.23 ± 5.76	− 0.5 ± 5.4	0.2 ± 5.6	0.2 ± 6.7

Means and SDs (in the format of *M* ± SD) for individual RT means, accuracy rates, and keypress durations, in Experiment 3a and in Experiment 3b; for *probe* (participants’ own countries), *irrelevant* (other countries), *target* (the designated irrelevant details that require different response), *P–I* (individual probe minus irrelevant values); per each filler-type condition (no filler, nonverbal, verbal)

*Exp.* Experiment, *T* target side, *NT* nontarget side

#### RT means

A one-way ANOVA, for probe-irrelevant RT mean differences, with the factor filler type with three levels (verbal, nonverbal, and no filler) was significant; *F*(2, 124) = 13.90, *p* < 0.001, *η*_p_^2^ = 0.183, 90% CI [0.084, 0.274], *η*_G_^2^ = 0.054, *BF*_*10*_ = 4468.91. As follow-up, we used three *t* tests for comparisons between each two of the three conditions. We were not interested in whether or not any of the conditions leads to decreased probe-irrelevant differences, as compared to the no filler version: Therefore, for the comparisons between no filler and nonverbal, and between no filler and verbal, we used one-sided *t* tests, expecting smaller RT mean differences in the no filler condition in both cases (as preregistered).

**Fig. 1:**
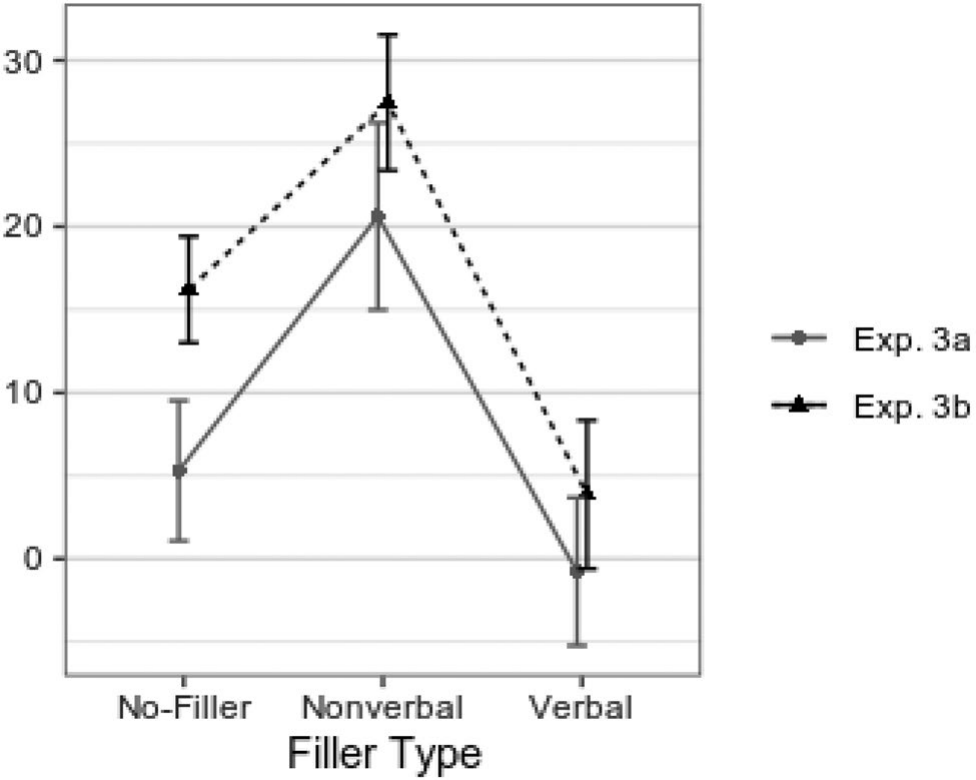
Probe-irrelevant RT differences per filler type. Means (with *SEM*s) of individual probe-irrelevant response time mean differences, in Experiments 3a and 3b; per each filler-type condition (no-filler, nonverbal, and verbal)

As expected, there were larger probe-irrelevant differences in the nonverbal condition than in the no filler condition, with very strong evidence and large nominal difference; *t*(62) = 3.51, *p* < 0.001, *d* = 0.44, 90% CI [0.22, ∞], *BF*_*10*_ = 60.81. However, contrary to our expectations, we found strong evidence for that the probe-irrelevant differences in the verbal condition are not larger than in the no filler condition; *t*(62) = 1.54, *p* = 0.935, *d* = 0.19, 90% CI [− ∞, 0.40], *BF*_*01*_ = 17.33. Finally, also somewhat contrary to expectations, probe-irrelevant differences in the nonverbal condition proved to be larger than in the verbal condition; *t*(62) = 5.08, *p* < 0.001, *d* = 0.64, 95% CI [0.37, 0.91], *BF*_*10*_ = 4558.69. The differences are depicted in Fig. 1 (along with similar results from Experiment 3b).

#### Accuracy rates

The one-way ANOVA, for probe-irrelevant accuracy rate differences, with the factor filler type, showed no significant differences; *F*(2, 124) = 1.90, *p* = 0.163, *ε* = 0.822, *η*_p_^2^ = 0.030, 90% CI [0, 0.084], *η*_G_^2^ = 0.017, *BF*_*01*_ = 3.32.

#### Keypress durations

Similarly, the one-way ANOVA, for probe-irrelevant keypress duration differences, with the factor filler type, showed no significant differences *F*(2, 124) = 2.90, *p* = 0.063, *ε* = 0.919, *η*_p_^2^ = 0.045, 90% CI [0, 0.107], *η*_G_^2^ = 0.029, *BF*_*01*_ = 1.19.

#### AUCs

The simulated AUCs for probe-irrelevant RT mean differences were 0.621, 95% CI [0.547, 0.694] for nonverbal, for 0.538, 95% CI [0.457, 0.618] no-filler, and 0.457, 95% CI [0.373, 0.541] for verbal.

### Interim discussion of Experiment 3a

While number string fillers (nonverbal condition) clearly led to the expected enhancement of probe-irrelevant RT differences, we found strong evidence for that the animate and inanimate concept fillers (verbal condition) do not lead to such enhancement. We had assumed that the verbal condition brings about a “highly increased task difficulty,” while the nonverbal condition brings about a “moderately increased task difficulty.” The present results might indicate that a moderate increase of difficulty indeed leads to enhancement, but a high increase of difficulty may be too much, and does not lead to enhancement. It might even be detrimental, although we did not test this latter assumption, since we used one-sided tests. The present results could likewise indicate that what matters more for the CIT effect is whether a categorical distinction of the fillers is systematically semantically related to the probe versus irrelevant distinction than if it invites elaboration of all items per se. For example, specific furniture and/or animal items may have had a higher relation to the self or to a particular country but whether such relations supported the CIT effect or not was then not systematically operationalized by the random assignments of animals versus furniture to the target versus irrelevant categories. It is also plausible that such putative self-relations were less obvious for digits and numbers, such that the nonverbal condition provided the better estimate of a true cognitive-load influence of the fillers on the CIT.

Therefore, we wanted to test if there is something particular about the specific items used here. This concerns particularly the verbal condition, which led to unexpected results. Hence, we decided to replicate Experiment 3a with different fillers, but with the same theoretical aim, and leaving all other settings unchanged.

The new verbal items were random pseudowords,^5^ to rule out any possible influence of the meaning of the items (yet at the same time leave the task relatively difficult, as pseudowords are relatively similar to real words and, therefore, require closer attention and semantic analysis; cf. Ratcliff, Gomez, & McKoon, 2004). Thus, we can rule out any connection to the self for these items and, thus, if a too high-task difficulty diminishes the CIT effect rather than enhances it, we expect again a weaker probe-irrelevant difference in this verbal condition in comparison to the nonverbal condition. The nonverbal items were in this case made even simpler, to demonstrate that they introduce only a comparatively lower (moderate) increase in task difficulty: We used random combinations of arrowhead-like symbols (unicode symbol characters), to be categorized with the key in the direction indicated by the symbols. For example, the item  had to be categorized with the response key on the left, and the item  had to be categorized with the response key on the right.

## Experiment 3b

### Methods

#### Participants

We initially opened 60 slots on Figure Eight, and 66 participants completed the task. Since the *BF* for the test between nonverbal and no-filler conditions did not reach 5, we opened 30 additional slots two times. Altogether 121 participants completed the test. Four participants had to be excluded due to too low accuracy rates, and six participants were excluded due to not recalling correctly the probes at the end of the task. This left 111 participants (*M*_age_ ± *SD*_age_ = 37.2 ± 12.7, 75 male).

#### Procedure

Same as in Experiment 3a, the main task, in each test, contained three blocks: one block with verbal fillers, one block with nonverbal fillers, and one with no fillers at all.

For the verbal condition, in each test, nine pseudowords were randomly selected from the following list (adapted from Hoversten, Brothers, Swaab, & Traxler 2017; originally generated using Wuggy: Keuleers & Brysbaert, 2010, and selected by native speakers): “angow,” “asheft,” “attish,” “bekish,” “bimality,” “boochamy,” “chalow,” “chathery,” “dakering,” “druckle,” “falward,” “fengaby,” “forpeat,” “immanick,” “lemrown,” “merfery,” “murtly, “ “nadwin,” “padgery,” “peetly,” “phernos,” “quath,” “reamuts,” “shurish,” “sprafty,” “stethery,” “tandly,” “truggy,” “unvethly,” “vismity,” “wheanory,” “whidical,” “wrintom.” The only restriction for the selection was that all selected pseudowords started with a unique letter (i.e., not the same starting letter as that of any of the other selected pseudowords). Analogously to previous fillers, three (randomly selected) pseudowords had to be categorized with the same key as the target, while the six other pseudowords had to be categorized with the other key. Again, all items were always displayed in uppercase.

In the Nonverbal block, the arrow-like fillers were items consisting of a number of different arrowhead-like unicode characters. For the three items to be categorized with the right-side Key *I* (also used for the target), the characters were randomly selected from among the following:  . For items to be categorized with the other, left-side Key *E*, the characters were randomly selected from among the following:  . (The random selection was again restricted in that each filler started with a unique character.) In either case, each such arrow-like filler had the same number of characters as the number of characters of a given matched verbal filler in the other block (for a filler categorized with the same key). For example, if a given participant had the pseudowords “attish,” “chathery,” and “quath” as verbal items to be categorized together with the target, they could have had, as nonverbal fillers to be categorized together with the target, the items  (or any other random variation using same numbers of symbols pointing to right).

### Results

Aggregated means of individual RT means, accuracy rates, and keypress durations, for the different stimulus types in each condition, are given in Table 3.

All tests (and indeed their outcomes, too) corresponded to those in Experiment 3a.

#### RT means

The one-way ANOVA, for probe-irrelevant RT mean differences, for the factor filler type with three levels (verbal, nonverbal, and no-filler) was significant (see Fig. 1); *F*(2, 220) = 15.72, *p* < 0.001, *ε* = 0.963, *η*_p_^2^ = 0.125, 90% CI [0.060, 0.190], *η*_G_^2^ = 0.051, *BF*_*10*_ = 3.22 × 10^4^. There were larger probe-irrelevant differences in the nonverbal condition than in the no-filler condition *t*(110) = 3.00, *p* = 0.002, *d* = 0.29, 90% CI [0.13, ∞], *BF*_*10*_ = 14.46. We again found strong evidence for probe-irrelevant differences in the verbal condition not being larger than in the no-filler condition; *t*(110) = 2.93, *p* = 0.998, *d* = 0.28, 90% CI [– ∞, 0.44], *BF*_*01*_ = 36.51. The probe-irrelevant differences in the nonverbal condition proved to be larger than in the verbal condition; *t*(110) = 5.11, *p* < 0.001, *d* = 0.49, 95% CI [0.29, 0.68], *BF*_*10*_ = 9961.96.

#### Accuracy rates

The one-way ANOVA, for probe-irrelevant accuracy rate differences, with the factor filler type, showed no significant differences; *F*(2, 220) = 2.23, *p* = 0.114, *ε* = 0.930, *η*_p_^2^ = 0.020, 90% CI [0, 0.054], *η*_G_^2^ = 0.010, *BF*_*01*_ = 3.97.

#### Keypress durations

Similarly, the one-way ANOVA, for probe-irrelevant keypress duration differences, with the factor filler type, showed no significant differences *F*(2, 220) = 0.48, *p* = 0.600, *ε* = 0.911, *η*_p_^2^ = 0.004, 90% CI [0, 0.023], *η*_G_^2^ = 0.003, *BF*_*01*_ = 18.49.

#### AUCs

The simulated AUCs for probe-irrelevant RT mean differences were 0.685, 95% CI [0.627, 0.743] for nonverbal, for 0.632, 95% CI [0.572, 0.692] no filler, and 0.495, 95% CI [0.429, 0.560] for verbal.

## General discussion

In the present investigation, we ran several experiments, with the goal to gain insight into the mechanism of filler items’ influence in the RT-CIT. Experiment 1 has shown that, as hypothesized, the smaller proportion of familiarity-related fillers leads to larger probe-irrelevant RT mean differences. Experiment 2 has shown that, as hypothesized, the semantic context of familiarity-related fillers, as well as their probe-incompatible key mapping significantly and robustly affects outcomes: When key mapping was reversed (familiarity-referring items categorized together with probe, instead of together with target), probe-irrelevant RT mean differences were on average reduced to almost zero. At the same time, both Experiments 1 and 2 provided evidence for that the visual distinctness of filler items from the other items does not affect the key outcomes (probe-irrelevant differences), at least when using such relatively minor visually distinguishing features (lettercase, underlining). In Experiments 3a and 3b (where 3b was a successful close conceptual replication of 3a), we found that using simple, symbolic stimuli (number strings or arrow-like characters), similarly to familiarity-related fillers, effectively increased probe-irrelevant differences, as hypothesized.

All in all, this demonstrates at least three largely independent factors that affect the RT-CIT: target to nontarget item proportion, semantic context, and task complexity. (Added to this, the target has its own separate role as well, demonstrated by Lukács & Ansorge, 2019a; see also Suchotzki, De Houwer, Kleinberg, & Verschuere 2018).

However, we also observed one finding that was completely unexpected: using, as fillers, words that are not systematically semantically relevant to the task (animate, inanimate concepts, or pseudowords) does not at all increase probe-irrelevant differences (but might even decrease them). This puzzling outcome (in both Experiment 3a and 3b) would require further experiments to be properly understood. Nonetheless, we here provide several comments and potential explanations that may help in the elucidation or at least provide hypotheses for future studies.

**Fig. 5:**
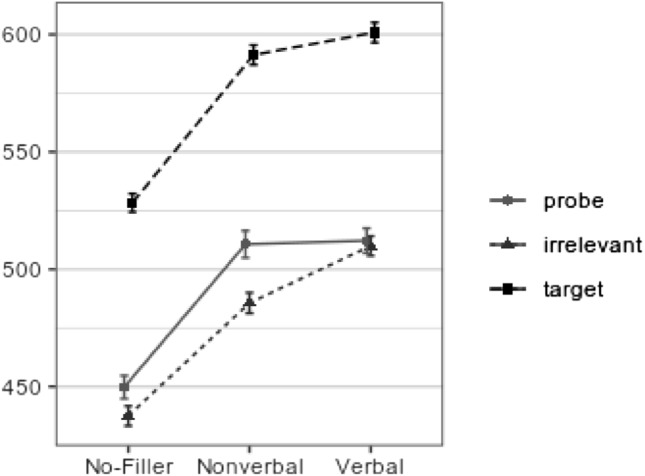
Response times for main items in Experiment 3. Means (with *SEM*s) of individual probe-irrelevant response time mean differences, in Experiment 3 (3a and 3b merged); per each filler-type condition (no-filler, nonverbal, and verbal)

**Fig. 6:**
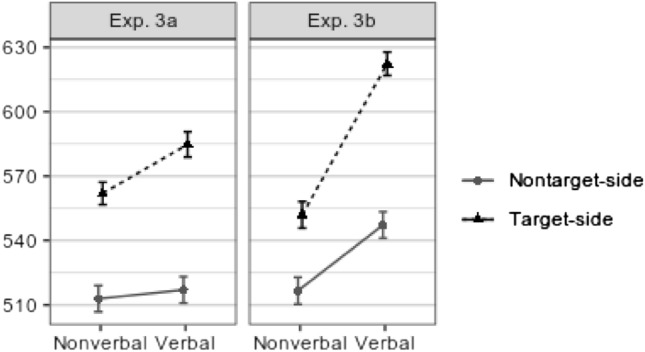
Response times for fillers in Experiment 3. Means (with *SEM*s) of individual probe-irrelevant response time mean differences for probes, in Experiments 3a and 3b; per filler-type conditions nonverbal and verbal, per filler key mapping (target side, nontarget side)

We assume that the main difference as compared with stimuli consisting of simple number or symbol characters (in the present Nonverbal condition) is that regular words are less easy to process quickly, and, hence, pose larger additional cognitive demand—as also implied by the generally lower accuracy rates and higher overall RTs in this condition (Table 3). It is possible that an overall increase of mean RTs for all items (in the verbal condition) masked the now less distinct response delay for probes (cf. Fiedler & Bluemke, 2005). Hence, one interpretation is that a certain level of increased task difficulty or complexity is beneficial (to foster probe response conflict and, thereby, increase probe-irrelevant differences), but too much can be detrimental, excessively draining cognitive resources, causing distraction (see also Lukács & Ansorge, 2019b, where probe-irrelevant differences were increased by reducing the unnecessary complexity of a specific CIT method), and allowing less interference through task-irrelevant information (cf. Lavie, Hirst, De Fockert, Viding, 2004). This explanation is also supported by the fact that the reduction of probe-irrelevant differences with verbal fillers was caused by a larger increase in irrelevant RTs as compared to the negligible increase in probe RTs—within the general pattern of RT increase for all item types from no-filler to nonverbal, and from nonverbal to verbal (Table 3; with *p* < 0.002 for all relevant comparisons in our exploratory tests in the Appendix; Figs. 5, 6).

Another possible explanation relates to the salience through rarity, as elaborated in the Introduction (in relation to filler proportions). Namely, the smaller, target-category portion of very easily distinguishable items, such as smaller or larger digits or, even more, left or right arrows, can be more easily regarded as a separate subgroup from the nontarget-category portion, while the same would not be true of object names, and, even more, of pseudowords (in verbal conditions).^6^ Thereby, the nonverbal target-category items would likely be more easily grouped by their rarity-based salience together with the targets and as opposed to the more frequent nontargets. As explained in the Introduction (and supported by the corresponding results of Experiment 1), target-category salience (here: by rarity of items across time) is likely to contribute to probe response conflict (Rothermund & Wentura, 2004). The striking opposition as in case of the arrow-like fillers that point left versus right might even help emphasize the subjective importance of the difference between the two response keys. That is, participants may have had a clearer notion that the two keys represent opposites, such as the oddball target versus the irrelevants, which, therefore, elicits larger response conflict for the oddball probe that is to be categorized together with the irrelevants.

Whether or not either of our proposed explanations is correct, an interesting question remains: how can familiarity-related fillers still be beneficial despite being meaningful words (which are presumed to add too much task difficulty)? First, it is possible that, since the fillers and their key mappings are semantically in line with the rest of the task, they are relatively easy to categorize (thereby, being similar to symbolic fillers), hence, do not pose excessive task difficulty. Second, it is possible, and perhaps even more likely, that familiarity-related fillers increase probe-irrelevant difference *despite* excessive task difficulty. This would mean that the beneficial effect of key mapping compatibility is so large that it towers over and masks the detrimental effect of excessive task difficulty, resulting in a significant beneficial net outcome on the CIT effect.

### Implications

The most straightforward implication is that the original rationale and structure of the fillers as these were introduced (Lukács et al., 2017b) is correct and optimal: the proportion of the fillers to be categorized together with the target should be kept low (Experiment 1), and the semantic key mapping of the fillers (if they are meaningful words) should be probe-incompatible (Experiment 2). This also indicates potential improvements in either case: The proportion could be even smaller (Hu et al., 2012; Suchotzki et al., 2015), and the filler could more specifically refer to the given probe (e.g. “MY COUNTRY” instead of “MINE”; Lukács et al., 2017a).

Furthermore, the present study demonstrates that the fillers can work in at least two separate ways: via semantics (Experiment 2) and via increased task complexity (via adding Nonverbal fillers; Experiments 3a and 3b). While we cannot say for sure how task complexity enhances the RT-CIT, the fact that simple, symbolic stimuli (number strings or arrow-like characters) also achieve enhancement has clear practical implications. The original familiarity-related fillers would need to be customized for each given scenario: for example, when the probe is a stolen property or the name or face of a suspected accomplice, we cannot use the filler “MINE” or “THEIRS,” etc., but we would need to find the appropriate probe-related filler words (e.g. “STOLEN” or “ACCOMPLICE”)—with each new set of fillers needing further empirical verification to ensure that they are optimal. Symbolic fillers are likely to be effective with any RT-CIT application regardless of scenario; that is, with any types of probe, target, and irrelevant items. They do not need to be translated to different languages, and they are unlikely to depend on the given writing system (see also below). They could even be used in cases where the suspect does not understand the examiners language well or even when the suspect is dyslexic or illiterate (assuming that also images or other easily recognizable probe, target, and irrelevant items can be found and used).

As explained above (regarding the finding with verbal fillers), we also suspect that excessive complexity may be detrimental. If this is true, this too indicates potential improvement in that one might be able to find an optimal task complexity to maximize probe-irrelevant RT differences. If it is not true, then, contrarily, one may further increase complexity (e.g., a third response key or more different items) to further enhance the RT-CIT—although we find this unlikely.

Finally, and again related to generalizability, the finding that visual distinctness has no substantial influence on the key CIT outcomes (at least when this distinctness is moderate) has two important implications. First, there is no need to visually match fillers to main items (e.g., in character length or starting letters). Consequently, there is much more room to choose any filler items, shifting emphasis to aim for proper semantic relevance (in case of fillers with probe-related meaning) and/or optimal task complexity. Conversely, there is also no need to make them distinct: if, for example, visual differences would have been found to be important, fillers would perform suboptimally in languages in which words are typically represented by single characters, with no salient visual differences. Second, perhaps the most imminent next step in this research direction is the use of fillers with images, with clear applied relevance in view of photographic evidence in forensic cases (Hsu, Lo, Ke, Lin, & Tseng 2020; Norman, Gunnell, Mrowiec, & Watson, 2020; Rosenfeld, Ward, Thai, & Labkovsky, 2015; Seymour & Kerlin, 2008). Tshe premise that visual distinctness has no modulating effect is promising given that images would be very distinct from textual filler words, and, hence, would be a concern otherwise.

### Summary

Supporting previously unproven assumptions, we demonstrated that the enhancing effects of filler items in RT-CIT depend on the proportion of filler categories, on the semantic relevance and semantic response key compatibility of fillers, and on the mere addition of fillers even if they are without relevant meaning, and we also gave evidence for that moderate visual distinctness of fillers has no significant effects. With this, we have given theoretical underpinning for the use of filler items, preliminary precepts for applied settings, and directions for further enhancements of the RT-CIT.

## Figures and Tables

**Fig. 2:**
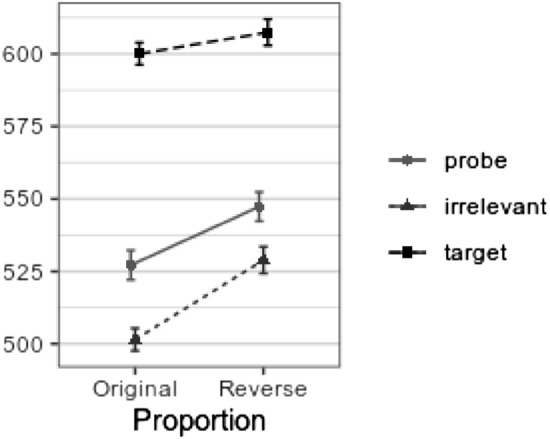
Response times for main items in Experiment 1. Means (with *SEM*s) of individual response time means for main items, in Experiment 1

**Fig. 3:**
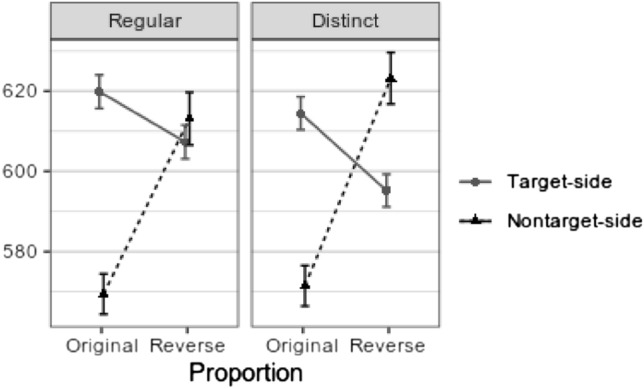
Response times for fillers in Experiment 1. Means (with *SEM*s) of individual response time means for fillers, in Experiment 1, per filler key mapping (target side, nontarget side)

**Fig. 4:**
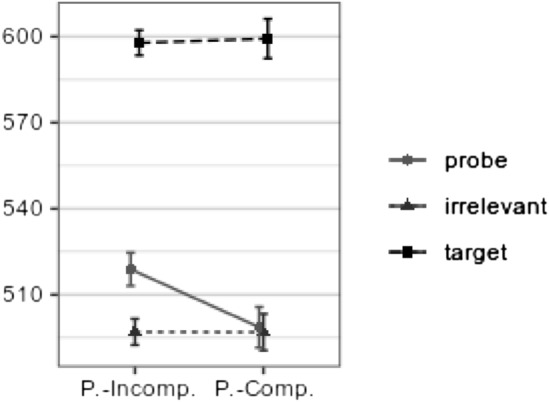
Response times for main items in Experiment 2. Means (with *SEM*s) of individual response time means, per main item type, in Experiment 2. *P.* probe, *Comp.* compatible, *Incomp.* incompatible

## Appendix: Supplementary tests

Here, we add a number of exploratory tests (mainly following the suggestions of a reviewer of an earlier version of this manuscript, J. P. Rosenfeld) that might be interesting for constructing further hypotheses.

For each experiment, we report ANOVAs, including Type factor that consists of probe and irrelevant RT means, and the factor(s) of the key manipulations of the given experiments. We separately report ANOVAs with Filler type factor that consists of target-category and nontarget-category filler RT means. We also provide corresponding figures, where appropriate, although these RT means per item types are also all shown in the tables in the main text.

## Experiment 1

### Main items

We conducted an ANOVA, for main item RT means, including the factors Type (probe, irrelevant), Proportion (Original, Reverse), and Distinctness (Regular, Distinct). A significant Type effect shows the usual higher RT means for probes, *F*(1, 132) = 58.37, *p* < 0.001, $\eta_{{\text{p}}}^{2}$ = 0.307, 90% CI [0.201, 0.400], $\eta_{{\text{G}}}^{2}$ = 0.036, *BF*_10_ = 4.89 × 10^18^. A significant Proportion main effect indicates higher RT means with Reverse proportion, *F*(1, 132) = 38.31, *p* < 0.001, $\eta_{{\text{p}}}^{2}$ = 0.225, 90% CI [0.127, 0.320], $\eta_{{\text{G}}}^{2}$ = 0.042, *BF*_10_ = 5.36 × 10^21^. The Proportion × Type interaction shows the larger probe-irrelevant difference with Original proportion, same as in the main text, *F*(1, 132) = 9.85, *p* = 0.002, $\eta_{{\text{p}}}^{2}$ = 0.069, 90% CI [0.016, 0.147], $\eta_{{\text{G}}}^{2}$ = 0.001, *BF*_01_ = 2.25 (Fig. ^2^). Nonetheless, probe RT means were significantly higher with both normal, *t*(132) = 7.63, *p* < 0.001, *d* = 0.66, 95% CI [0.47, 0.85], *BF*_10_ = 1.90 × 10^9^, and reversed proportions, *t*(132) = 6.51, *p* < 0.001, *d* = 0.56, 95% CI [0.38, 0.75], *BF*_10_ = 6.51 × 10^6^.

Distinct fillers lead to generally lower RT means, *F*(1, 132) = 13.40, *p* < 0.001, $\eta_{{\text{p}}}^{2}$ = 0.092, 90% CI [0.028, 0.175], $\eta_{{\text{G}}}^{2}$ = 0.007, *BF*_10_ = 301.53 (Table 1 in main text), but no significant interactions; distinctness × proportion: *F*(1, 132) = 0.27, *p* = 0.604, $\eta_{{\text{p}}}^{2}$ = 0.002, 90% CI [0, 0.033], $\eta_{{\text{G}}}^{2}$ < 0.001, *BF*_01_ = 11.08; distinctness × type: *F*(1, 132) = 0.73, *p* = 0.396, $\eta_{{\text{p}}}^{2}$ = 0.005, 90% CI [0, 0.044], $\eta_{{\text{G}}}^{2}$ < 0.001, *BF*_01_ = 4.69; distinctness × proportion × type: *F*(1, 132) = 2.40, *p* = 0.123, $\eta_{{\text{p}}}^{2}$ = 0.018, 90% CI [0, 0.071], $\eta_{{\text{G}}}^{2}$ < 0.001, *BF*_01_ = 5.60.

### Fillers

We conducted an ANOVA, for filler RT means, including the factors filler-side (i.e., filler key mapping: target side or nontarget side), proportion (original, reverse), and distinctness (regular, distinct), see Fig. 3. A significant filler-side effect indicated higher RT means for target-side fillers, *F*(1, 132) = 16.04, *p* < 0.001, $\eta_{{\text{p}}}^{2}$ = 0.108, 90% CI [0.038, 0.195], $\eta_{{\text{G}}}^{2}$ = 0.016, *BF*_10_ = 4.18 × 10^5^, although the distinctness × filler-side interaction indicates that this difference was smaller with distinct fillers, *F*(1, 132) = 25.66, *p* < 0.001, $\eta_{{\text{p}}}^{2}$ = 0.163, 90% CI [0.077, 0.255], $\eta_{{\text{G}}}^{2}$ = 0.004, *BF*_10_ = 9.66. A significant proportion main effect again indicates higher RT means with reverse proportion, *F*(1, 132) = 21.00, *p* < 0.001, $\eta_{{\text{p}}}^{2}$ = 0.137, 90% CI [0.058, 0.228], $\eta_{{\text{G}}}^{2}$ = 0.018, *BF*_10_ = 3.50 × 10^6^. The distinctness main effect here is not significant, *F*(1, 132) = 0.38, *p* = 0.537, $\eta_{{\text{p}}}^{2}$ = 0.003, 90% CI [0, 0.036], $\eta_{{\text{G}}}^{2}$ < 0.001, *BF*_01_ = 13.52, and this was not influenced by the proportion factor, *F*(1, 132) = 0.04, *p* = 0.840, $\eta_{{\text{p}}}^{2}$ < 0.001, 90% CI [0, 0.017], $\eta_{{\text{G}}}^{2}$ < 0.001, *BF*_01_ = 11.67.

The proportion × filler-side interaction indicates a robust modulation of the filler filler-side effect: with original proportion, target-side fillers have higher RT means, while with reverse proportion, nontarget-side fillers have higher RT means, *F*(1, 132) = 288.61, *p* < 0.001, $\eta_{{\text{p}}}^{2}$ = 0.686, 90% CI [0.614, 0.736], $\eta_{{\text{G}}}^{2}$ = 0.070, *BF*_10_ = 1.80 × 10^33^. This interaction itself was modulated by the distinctness factor: the three-way interaction (distinctness × proportion × filler side) indicates that the proportion × filler-side interaction is larger when fillers are distinct (as can be seen on Fig. 3), *F*(1, 132) = 8.63, *p* = 0.004, $\eta_{{\text{p}}}^{2}$ = 0.061, 90% CI [0.012, 0.137], $\eta_{{\text{G}}}^{2}$ = 0.001, *BF*_01_ = 2.01 (indicating that the experimental manipulation was successful.).

## Experiment 2

### Main items

We conducted an ANOVA, for RT means, including the factors (main item) type (probe, irrelevant) and semantic context (probe incompatible and probe compatible). Due to violations of sphericity (Mauchly's sphericity test: *W* = 0.746, *p* < 0.001), Huynh–Feldt correction is applied to the type main effect and type × semantic context interaction tests.

A significant type effect again shows the usual higher RT means for probes, *F*(2, 404) = 782.36, *p* < 0.001, ε = 0.803, $\eta_{{\text{p}}}^{2}$ = 0.795, 90% CI [0.768, 0.815], $\eta_{{\text{G}}}^{2}$ = 0.377, *BF*_10_ = 3.86 × 10^133^, while the semantic context factor shows no significant difference, *F*(1, 202) = 0.59, *p* = 0.442, $\eta_{{\text{p}}}^{2}$ = 0.003, 90% CI [0, 0.028], $\eta_{{\text{G}}}^{2}$ = 0.002, *BF*_01_ = 3.75. The type × semantic context interaction shows larger difference between probe and irrelevant RTs (same as in the main text), *F*(2, 404) = 7.92, *p* = 0.001, ε = 0.803, $\eta_{{\text{p}}}^{2}$ = 0.038, 90% CI [0.011, 0.070], $\eta_{{\text{G}}}^{2}$ = 0.006, *BF*_10_ = 54.13 (Fig. 4). Probe RT means were higher than irrelevant RT means in the probe-incompatible condition, *t*(144) = 7.57, *p* < 0.001, *d* = 0.63, 95% CI [0.45, 0.81], *BF*_10_ = 1.88 × 10^9^, but not in the probe-compatible condition, *t*(58) = 0.50, *p* = 0.620, *d* = 0.06, 95% CI [− 0.19, 0.32], *BF*_01_ = 6.24.

Apparently, as opposed to the probe, neither the target nor the irrelevants were affected by semantic context (i.e., fillers mapped to probe-compatible keys). Our explanation is that the probe is by far the most sensitive to the influence of fillers because that is the single item that is strongly related to the semantic context induced by the fillers (compatible familiarity, self-relatedness vs. incompatible unfamiliarity, other-relatedness). The irrelevants are neither familiar (self-related), nor explicitly “unfamiliar” (other-related), that is, they are unlikely to be strongly associated with the concept of “unfamiliarity” (other-relatedness). Similarly, the target itself is not in fact truly “familiar” (and certainly not self-related, such as indicated by the filler “MINE”) to the participants, and, therefore, responses to it are unlikely to be substantially affected by the semantic context induced by fillers.

Nonetheless, it is also quite possible that with more participants and more controlled settings, the target and irrelevant items might also show clearer changes: the lack of substantial changes certainly indicates that these items are not as much affected as the probe, but it cannot be said for certain that they are not affected at all. In any case, this issue could be explored further, but from the practical perspective the most important finding is clear: The semantic context induced by the fillers plays a decisive role in enhancing probe-irrelevant differences.

### Fillers

For filler RTs, we conducted an ANOVA, including the factors filler side and semantic context. A significant filler-side effect again indicated higher RT means for target-side fillers, *F*(1, 202) = 496.64, *p* < 0.001, $\eta_{{\text{p}}}^{2}$ = 0.711, 90% CI [0.658, 0.750], $\eta_{{\text{G}}}^{2}$ = 0.203, *BF*_10_ = 3.07 × 10^52^. Neither the semantic context main effect nor the semantic context × filler-side interaction was significant, *F*(1, 202) = 0.08, *p* = 0.778, $\eta_{{\text{p}}}^{2}$ < 0.001, 90% CI [0, 0.015], $\eta_{{\text{G}}}^{2}$ < 0.001, *BF*_01_ = 3.32., *F*(1, 202) = 1.66 (main effect), *p* = 0.199, $\eta_{{\text{p}}}^{2}$ = 0.008, 90% CI [0, 0.040], $\eta_{{\text{G}}}^{2}$ = 0.001, *BF*_01_ = 2.73 (interaction).

## Experiment 3

### Main items

For probe and irrelevant analysis, participants from Experiment 3a and 3b are merged together, for simplicity. The same tests for each Experiment lead to analogous results, and taking “Experiment” as an additional factor into the ANOVA gives no significant results: *p* > 0.13 for the main effect and all interactions.

We conducted an ANOVA, for RT means, including the factors (main item) Type (probe, irrelevant) and filler type (verbal, nonverbal, and no-filler). Due to violation of sphericity (*W* = 0.943, *p* = 0.006), Huynh–Feldt correction is applied to the Filler Type main effect.

A significant type effect once again shows the usual higher RT means for probes, *F*(1, 173) = 27.66, *p* < 0.001, $\eta_{{\text{p}}}^{2}$ = 0.138, 90% CI [0.067, 0.217], $\eta_{{\text{G}}}^{2}$ = 0.011, *BF*_10_ = 1.72 × 10^5^. A significant filler type effect indicates that RTs differ depending on filler type, *F*(2, 346) = 183.23, *p* < 0.001, ε = 0.956, $\eta_{{\text{p}}}^{2}$ = 0.514, 90% CI [0.455, 0.562], $\eta_{{\text{G}}}^{2}$ = 0.174, *BF*_10_ = 7.68 × 10^95^. The type × filler type interaction shows that the difference between probe and irrelevant RTs also depends on filler type, *F*(2, 346) = 27.52, *p* < 0.001, $\eta_{{\text{p}}}^{2}$ = 0.137, 90% CI [0.083, 0.191], $\eta_{{\text{G}}}^{2}$ = 0.005, *BF*_10_ = 30.64 (Fig. 5).

To decompose the 2 by 3 interaction, the ANOVA was repeated for each of the three possible pairs of filler type (i.e., always leaving out one). The ANOVA with no-filler and nonverbal (but without verbal) shows that probe RTs are larger than irrelevant RTs (type main effect), *F*(1, 173) = 51.63, *p* < 0.001, $\eta_{{\text{p}}}^{2}$ = 0.230, 90% CI [0.143, 0.313], $\eta_{{\text{G}}}^{2}$ = 0.021, *BF*_10_ = 3.13 × 10^7^, RTs with nonverbal fillers are larger than with no fillers (filler-type main effect), *F*(1, 173) = 185.68, *p* < 0.001, $\eta_{{\text{p}}}^{2}$ = 0.518, 90% CI [0.434, 0.583], $\eta_{{\text{G}}}^{2}$ = 0.154, *BF*_10_ = 2.19 × 10^57^, and the interaction shows that the difference between probes and irrelevants is larger with nonverbal fillers, *F*(1, 173) = 19.75, *p* < 0.001, $\eta_{{\text{p}}}^{2}$ = 0.102, 90% CI [0.041, 0.177], $\eta_{{\text{G}}}^{2}$ = 0.002, *BF*_10_ = 1.13.

The ANOVA with no-filler and verbal (but without nonverbal) shows that probe RTs are larger than irrelevant RTs (type main effect), *F*(1, 173) = 8.24, *p* = 0.005, $\eta_{{\text{p}}}^{2}$ = 0.045, 90% CI [0.008, 0.105], $\eta_{{\text{G}}}^{2}$ = 0.003, *BF*_10_ = 2.14, RTs with Verbal fillers are larger than with no fillers (filler-type main effect), *F*(1, 173) = 297.87, *p* < 0.001, $\eta_{{\text{p}}}^{2}$ = 0.633, 90% CI [0.562, 0.684], $\eta_{{\text{G}}}^{2}$ = 0.230, *BF*_10_ = 1.68 × 10^81^, and the interaction shows that the difference between probes and irrelevants is larger in no-filler conditions, *F*(1, 173) = 10.91, *p* = 0.001, $\eta_{{\text{p}}}^{2}$ = 0.059, 90% CI [0.015, 0.124], $\eta_{{\text{G}}}^{2}$ = 0.002, *BF*_01_ = 2.13.

Analogously, the ANOVA with nonverbal and verbal (but without no-filler condition) shows that probe RTs are larger than irrelevant RTs (type main effect), *F*(1, 173) = 22.90, *p* < 0.001, $\eta_{{\text{p}}}^{2}$ = 0.117, 90% CI [0.051, 0.193], $\eta_{{\text{G}}}^{2}$ = 0.011, *BF*_10_ = 1.41 × 10^4^, RTs with verbal fillers are larger than with no fillers (filler type main effect), *F*(1, 173) = 15.60, *p* < 0.001, $\eta_{{\text{p}}}^{2}$ = 0.083, 90% CI [0.028, 0.153], $\eta_{{\text{G}}}^{2}$ = 0.010, *BF*_10_ = 3876.29, and the interaction shows that the difference between probes and irrelevants is larger with nonverbal fillers, *F*(1, 173) = 47.45, *p* < 0.001, $\eta_{{\text{p}}}^{2}$ = 0.215, 90% CI [0.131, 0.299], $\eta_{{\text{G}}}^{2}$ = 0.008, *BF*_10_ = 630.47.

To sum up the conclusions of the decomposition (cf. Fig. 5): (1) overall RTs were smallest with no filler, larger with nonverbal, largest with verbal, and (2) the difference between probe and irrelevant RTs was smallest with verbal, larger with no filler, largest with nonverbal (Fig. 6).

Finally, pairwise comparisons per filler type show that probe RT means were significantly higher than irrelevant RT means with nonverbal and verbal fillers, *t*(173) = 7.56, *p* < 0.001, *d* = 0.57, 95% CI [0.41, 0.73], *BF*_10_ = 3.25 × 10^9^ (nonverbal), *t*(173) = 4.76, *p* < 0.001, *d* = 0.36, 95% CI [0.21, 0.51], *BF*_10_ = 3073.20 (no filler), but not with verbal fillers, *t*(173) = 0.67, *p* = 0.504, *d* = 0.05, 95% CI [− 0.10, 0.20], *BF*_01_ = 9.49.

### Fillers

The final ANOVA, for filler RTs, includes the factors filler category (target category, nontarget category), filler type (nonverbal or verbal), and Experiment (3a, 3b). The significant filler-category main effect again indicates higher RT means for target-category fillers, *F*(1, 172) = 394.21, *p* < 0.001, $\eta_{{\text{p}}}^{2}$ = 0.696, 90% CI [0.636, 0.740], $\eta_{{\text{G}}}^{2}$ = 0.193, *BF*_10_ = 3.02 × 10^63^. The significant filler-type main effect indicates higher RTs in case of verbal fillers, *F*(1, 172) = 137.55, *p* < 0.001, $\eta_{{\text{p}}}^{2}$ = 0.444, 90% CI [0.354, 0.517], $\eta_{{\text{G}}}^{2}$ = 0.094, *BF*_10_ = 2.31 × 10^29^. The significant Experiment main effect indicates higher RT means in Experiment 3b, *F*(1, 172) = 3.96, *p* = 0.048, $\eta_{{\text{p}}}^{2}$ = 0.023, 90% CI [0.000, 0.071], $\eta_{{\text{G}}}^{2}$ = 0.016, *BF*_10_ = 1.14. The experiment × filler-type interaction indicates larger differences between nonverbal and verbal fillers in Experiment 3b, *F*(1, 172) = 31.60, *p* < 0.001, $\eta_{{\text{p}}}^{2}$ = 0.155, 90% CI [0.080, 0.236], $\eta_{{\text{G}}}^{2}$ = 0.023, *BF*_10_ = 6.56 × 10^7^. The filler type × filler-side interaction indicates larger differences between target-side and nontarget-side fillers in case of verbal fillers, *F*(1, 172) = 66.83, *p* < 0.001, $\eta_{{\text{p}}}^{2}$ = 0.280, 90% CI [0.189, 0.363], $\eta_{{\text{G}}}^{2}$ = 0.019, *BF*_10_ = 1.62 × 10^6^. The experiment × filler-side interaction is not significant, *F*(1, 172) = 0.29, *p* = 0.593, $\eta_{{\text{p}}}^{2}$ = 0.002, 90% CI [0, 0.026], $\eta_{{\text{G}}}^{2}$ < 0.001, *BF*_01_ = 6.32. Finally, the experiment × fillertype × filler side three-way interaction indicates that the fillertype × filler-side interaction (see above) is larger in Experiment 3b, *F*(1, 172) = 6.69, *p* = 0.011, $\eta_{{\text{p}}}^{2}$ = 0.037, 90% CI [0.005, 0.094], $\eta_{{\text{G}}}^{2}$ = 0.002, *BF*_01_ = 1.22.

## Footnotes

1 It has been argued that the target shares the feature of “familiarity” with the probe, which may be the cause of the probe response conflict (Verschuere & De Houwer, 2011). However, the target is not actually “familiar” to examinees in the real sense of the word: It is randomly selected from among the irrelevant items and (thus arbitrarily) designated as requiring a different response. (See also the Main Items section under Experiment 2 in the Appendix.).

2 As we were interested in understanding the modulating influence of fillers on CIT effects, only the fillers were manipulated. However, we understand that in this way, subjects were not spared an additional discrimination beyond a visual one between fillers of the different response categories. Thus, here, we rather tested if grouping of targets and their related fillers on the one hand, and of irrelevants and probes and their related fillers on the other hand, was a modulating factor for the CIT effect.

3 This was not preregistered but it seemed reasonable because, depending on the interstimulus interval, the maximum possible recorded duration randomly varied between 200 and 500 ms, hence, we can only know the duration for certain when it was less than 200 ms. In any case, (a) the vast majority of keypress durations are below 200 ms (see the means and standard deviations, in e.g., Table 1), (b) this only concerns the secondary analysis of keypress durations, and (c) this criterion makes no relevant changes to the outcomes.

4 As can be seen from the degrees of freedom, three participants had to be excluded because they had too few valid key presses for at least one of the item types in at least one of the blocks. This can happen (despite otherwise having sufficient valid responses for the other analyses) due to the exclusion of keypress durations not below 200 ms. There were two similarly excluded participants in Experiment 2, and none in Experiments 3a and 3b.

5 The term “verbal” is actually not entirely accurate for describing pseudowords. A more linguistically precise designation of the two conditions in both Experiment 3a and 3b would be “graphemic-syllabic” and “non-graphemic.” We nonetheless decided by the terms “verbal” and “nonverbal,” because they are simpler and more commonly understood.

6 This perspective was first proposed by Olson et al. (2020), as “chunking,” inspiring the present paragraph—although our general interpretation of how this influences the task is somewhat different.
